# Effects of Arterial Carbon Dioxide Tension on Cerebral and Somatic Regional Tissue Oxygenation and Blood Flow in Neonates After the Norwood Procedure With Deep Hypothermic Cardiopulmonary Bypass

**DOI:** 10.3389/fped.2022.762739

**Published:** 2022-02-11

**Authors:** George M. Hoffman, John P. Scott, Eckehard A. Stuth

**Affiliations:** ^1^Division of Pediatric Cardiac Anesthesia, Children's Hospital of Wisconsin Herma Heart Institute, Milwaukee, WI, United States; ^2^Division of Pediatric Cardiac Critical Care, Children's Hospital of Wisconsin Herma Heart Institute, Milwaukee, WI, United States; ^3^Department of Anesthesiology, Medical College of Wisconsin, Milwaukee, WI, United States; ^4^Department of Pediatrics, Medical College of Wisconsin, Milwaukee, WI, United States

**Keywords:** NIRS (near infrared reflectance spectroscopy), cerebral oxygenation, regional blood flow, somatic oxygenation, carbon dioxide, neonatal brain, cerebral autoregulation

## Abstract

Neonates undergoing the Norwood procedure for hypoplastic left heart syndrome are at higher risk of impaired systemic oxygen delivery with resultant brain, kidney, and intestinal ischemic injury, shock, and death. Complex developmental, anatomic, and treatment-related influences on cerebral and renal-somatic circulations make individualized treatment strategies physiologically attractive. Monitoring cerebral and renal circulations with near infrared spectroscopy can help drive rational therapeutic interventions. The primary aim of this study was to describe the differential effects of carbon dioxide tension on cerebral and renal circulations in neonates after the Norwood procedure. Using a prospectively-maintained database of postoperative physiologic and hemodynamic parameters, we analyzed the relationship between postoperative arterial carbon dioxide tension and tissue oxygen saturation and arteriovenous saturation difference in cerebral and renal regions, applying univariate and multivariate multilevel mixed regression techniques. Results were available from 7,644 h of data in 178 patients. Increases in arterial carbon dioxide tension were associated with increased cerebral and decreased renal oxygen saturation. Differential changes in arteriovenous saturation difference explained these effects. The cerebral circulation showed more carbon dioxide sensitivity in the early postoperative period, while sensitivity in the renal circulation increased over time. Multivariate models supported the univariate findings and defined complex time-dependent interactions presented graphically. The cerebral and renal circulations may compete for blood flow with critical limitations of cardiac output. The cerebral and renal-somatic beds have different circulatory control mechanisms that can be manipulated to change the distribution of cardiac output by altering the arterial carbon dioxide tension. Monitoring cerebral and renal circulations with near infrared spectroscopy can provide rational physiologic targets for individualized treatment.

## Introduction

The neonate undergoes profound changes in pulmonary and systemic circulations over the first week of life, with overlying developmental influences on circulatory controls of cerebral and renal-mesenteric systemic beds (1–4). While both local autoregulatory mechanisms and sympathetic nervous system modulation of regional resistance are present throughout the systemic circulation, the differential physiologic controls over cerebral and somatic regions result from profoundly different magnitudes of these mechanisms. In addition to vulnerabilities related to the transitional circulation and the neonatal myocardium, neonates with univentricular heart disease have vulnerabilities related to decreased ventricular mass, obligate mixing with arterial desaturation, and the pulmonary-systemic tradeoff inherent in parallel circulation (5–8). Such circulatory limitations increase the risk for hypoxic-ischemic injury in both cerebral and renal/somatic circulations (3, 9).

In neonates with univentricular parallel circulatory anatomy, the various systemic regional circulations compete with each other and with the pulmonary circulation. Monitoring cerebral and renal/somatic circulations with near-infrared spectroscopy (NIRS) in neonates before, during, and after surgical palliation of hypoplastic left heart syndrome (HLHS) with the Norwood procedure, has helped uncover circulatory vulnerabilities, describe relationships between monitored parameters and outcomes, and determine targets for intervention (3, 8, 10–14). Both cerebral and renal-somatic organ beds are at risk for hypoxic injury following Norwood palliation of HLHS (9, 11, 15–21). Hypothermic cardiopulmonary bypass produces significant postoperative changes in cerebrovascular resistance (3, 22–27). Resulting early postoperative cerebral desaturation has been linked to later neurodevelopmental delays (11, 28–30). Perioperative renal/somatic desaturation has been associated with acute kidney injury as well as necrotizing enterocolitis (31–33). The combination of arterial blood pressure and renal oxygenation by NIRS can define a low cardiac output condition associated with increased mortality (13).

Modulation of systemic and pulmonary vascular resistance with induced changes in arterial carbon dioxide tension has been utilized to affect systemic-pulmonary balance, with particularly prominent effects noted on the cerebral circulation (34–37). In univentricular parallel circulation, deliberate modulation of regional resistances may be helpful to avoid end-organ injury, since total oxygen delivery may be critically limited (5, 38, 39). In this report, we describe the complex effects of carbon dioxide on systemic cerebral and renal regional circulations in neonates following surgical palliation of HLHS with a Norwood procedure.

## Materials and Methods

The patient population included neonates with HLHS undergoing a Norwood procedure for arch reconstruction and systemic to pulmonary shunt placement in the first month of life. Patients underwent preoperative stabilization with prostaglandin infusion, and treatment with sedatives, inotropic-vasoactive infusions, and mechanical ventilation as necessary. Monitoring included continuous assessment of arterial oxyhemoglobin saturation (SaO2) by pulse oximetry, mean arterial pressure (MABP) transduced from an umbilical or radial artery catheter, central venous or atrial pressure from an umbilical venous or atrial catheter, and regional tissue oxyhemoglobin saturation by NIRS with probes over the midline-right forehead and right T12 to L2 flank regions for cerebral (rSO2C) and renal-somatic (rSO2R) fields, respectively. Surgical procedures were performed with deep hypothermic cardiopulmonary bypass (CPB) at 18–20 degrees Celsius with pH stat blood gas management, alpha-adrenergic blockade (AAB), and with selective antegrade cerebral perfusion (ACP) to limit duration of deep hypothermic circulatory arrest (DHCA), under high dose opioid based volatile supplemented anesthesia (3, 13, 40–42). Infusions of milrinone, epinephrine, and norepinephrine were titrated prior to weaning from CPB to achieve a calculated systemic vascular resistance index of about 12 Wood units. Physiologic targets following cardiopulmonary bypass included SaO2 75–85%, MABP > 50 mmHg, rSO2C > 50%, rSO2R > 60%. Patients were recovered in the cardiac ICU with continuation of opioid and vasoactive infusions, red blood cell transfusion, elective mechanical ventilation, controlled normothermia, and planned delayed sternal closure to achieve these physiologic goals.

Demographic surgical physiologic and hemodynamic data were collected at hourly intervals for the first post-operative 48 h following the Norwood procedure and were stored in a clinical registry with IRB approval. The primary physiologic outcome measures were cerebral (rSO2C) and renal (rSO2R) field saturations, regional venous saturations (SvO2C, SvO2R), and arterio-venous differences (ΔSavO2C, ΔSavO2R) calculated from simultaneous measures using a 25%/75% arterial/venous field saturation model. Arterial blood samples were obtained at standard intervals with linear interpolation for values synchronous with hourly recordings of physiologic monitoring data. Details of measures and derivations are shown in Table 1. Physiologic data were excluded from analysis during periods of extracorporeal circulatory support. Data were summarized as mean plus or minus standard deviation, median and interquartile range and 5–95% confidence intervals.

**Table 1:** Sources and formulae for measured and derived physiologic variables.

**Variable**	**Definition**	**Source or derivation**
SaO2	Arterial oxyhemoglobin saturation	Masimo SET
rSO2C rSO2R	Regional tissue saturation (cerebral field) Regional tissue saturation (renal-somatic field)	Medtronics/Somanetics Invos 5100A pediatric algorithm
SvO2C SvO2R	Regional venous saturation (cerebral) Regional venous saturation (renal)	SvO2C = (rSO2C−0.25*SaO2)/0.75 SvO2R = (rSO2R−0.25*SaO2)/0.75
ΔSavO2C ΔSavO2R	Regional arteriovenous difference (cerebral) Regional arteriovenous difference (renal)	ΔSavO2C = SaO2 – SvO2C ΔSavO2R = SaO2 – SvO2R
ΔSarSO2C ΔSarSO2R	Regional arterial-tissue difference (cerebral) Regional arterial-tissue difference (renal)	ΔSarO2C = SaO2 – rSO2C ΔSarO2R = SaO2 – rSO2R
fOERvC fOERvR	Regional venous extraction ratio (cerebral) Regional venous extraction ratio (renal)	fOERvC = ΔSavO2C/SaO2 fOERvR = ΔSavO2R/SaO2
fOERrC fOERrR	Regional tissue extraction ratio (cerebral) Regional tissue extraction ratio (renal)	fOERrC = ΔSarO2C/SaO2 fOERrR = ΔSarO2R/SaO2
MABP CVP HR	Mean arterial blood pressure Mean venous or atrial pressure Heart rate	GE Solar
PaCO2	Arterial carbon dioxide	Radiometer ABL
PaO2	Arterial oxygen tension	
pH	Negative log hydrogen ion	
Hgb	Hemoglobin concentration	

Univariable fixed-effects models were used to test association of the primary variables regional field saturations and arteriovenous differences with linear and non-linear components for PaCO2 and postoperative time. Multivariable mixed effect models were then used to define associations of primary variables with interactions of PaCO2 and time, using other demographic, treatment, and physiologic measures as covariates. The combined effects of PaCO2 and time on the primary physiologic outcome measures were derived from these models with defined values for covariates and displayed in graphical form. Finally, the adjusted marginal effects of changes in PaCO2 on outcome measures were calculated for each postoperative hour. All models used cross-sectional panel regression methods to allow within-patient and between-patient variance specifications robust to non-independent clustering and non-normality. Model coefficients were expressed with point estimate ± standard error, with significant effects identified at *p* < 0.05 (^*^), *p* < 0.01 (^**^), and *p* < 0.001 (^***^) levels. Analyses were performed using Stata Software (v17, RRID:SCR_012763) and Python Programming Language (v3.8, RRID:SCR_008394).

## Results

### Population

The study population was derived from 195 neonates with postoperative cerebral and renal NIRS monitoring, with operative survival in 176 (90.3%). Data were analyzable from 178 patients after exclusion of 17 patients requiring continuous ECMO support in the first 48 h following operative intervention. This analysis population had slightly higher weight at operation, with less total CPB support time, with differences shown in Supplementary Table S1. Survival in the analysis population was 94%, with ECMO used in 2% of survivors vs. 30% of non-survivors Complete population demographic and operative characteristics are shown in Table 2.

**Table 2:** Patient population demographics, operative characteristics, and outcomes.

**Characteristic**	**All**	**Survived**	**Died**	***P-value***
N (%)	178 (100%)	168 (94%)	10 (5.6%)	
Weight at S1P (kg)	3.20 (0.70) (2.90–3.60)	3.20 (0.71) (2.90–3.61)	3.15 (1.04) 2.52–3.56)	0.368
Age at S1P (days)	7.0 (4.0) (5.0–9.0)	7.00 (4.00) (5.0–9.0)	9.50 (6.00) (5.5–11.5)	0.121
Gender: Male Female	104 (58%) 74 (42%)	100 (60%) 68 (40%)	4 (40%) 6 (60%)	0.224
Gestational age (weeks)	38.0 (1.0) (38.0–39.0)	38.0 (1.0) (38.0–39.0)	38.0 (1.8) (37.0–38.8)	0.306
CPB time (minutes)	168 (44) (148–192)	168 (43) (148–191)	168 (88) (160–248)	0.213
DHCA time (minutes)	12 (8) (8–16)	12 (8) (8–16)	12 (15) (7–22)	0.702
Shunt type: MBTS RVPA	95 (53%) 83 (47%)	91 (54%) 77 (46%)	4 (40%) 6 (60%)	0.383
ECMO support yes No	7 (4%) 171 (96%)	4 (2%) 164 (98%)	3 (30%) 7 (70%)	<0.001
Hospital LOS (days)	38 (38) (28–66)	38 (34) (28–62)	52 (85) (25–111)	0.795

*Data are presented as median (interquartile range) and interval or count (percent). Data were excluded from analysis during ECMO and operative support, yielding an analysis set of 178 patients, with 168 (94%) operative survival through hospital discharge. Differences between survivors and non-survivors are summarized by significance testing*.

### Summary Statistics and Variation

The analysis set comprised 7,644 h of data with an average of 43 h per patient. Postoperative hemodynamic state was characterized by mean SaO2 83.0 ± 4.9%, MABP 49.8 ± 5.4 mmHg, CVP 9.6 ± 2.2 mmHg, and hemoglobin 16 ± 1.5 gm/dl. Regional saturations were rSO2C 65.9 ± 8.6% and rSO2R 75.9 ± 8.7%, with arteriovenous differences ΔSavO2C 23.3 ± 11.2% and ΔSavO2R 9.4 ± 11.6%. The SaO2 had the lowest overall variability, with regional saturations rSO2C and rSO2R nearly twice as variable. The measures of regional flow (arteriovenous differences and fractional extraction) showed the greatest overall variation. Variance partitioning reve aled a range of within-patient and between-patient variance components. Measures of PaCO2 and cerebral oxygenation and flow showed the most between-patient variation, while SaO2 showed the most within-patient variability, followed by renal arteriovenous difference. Details of physiologic parameters including components of deviation between and within patients are shown in Table 3.

**Table 3:** Summary of physiologic parameters.

**Variable**	**P50**	**IQR**	**P5**	**P95**	**SK**	**Mean**	**SD**	**SD(W)**	**SD(B)**	**(W/B)**	**CV**	**CV(W)**
SaO2	83.0	6.0	75.0	91.0	−0.43	83.0	4.9	3.8	3.4	1.11	0.06	0.07
rSO2C	66.0	11.0	51.0	79.0	−0.24	65.9	8.6	5.6	7.6	0.79	0.13	0.10
rSO2R	77.0	11.0	60.0	89.0	−0.56	75.9	8.7	5.9	6.7	0.89	0.11	0.10
SvO2C	60.3	14.3	40.7	77.0	−0.25	59.8	11.0	7.2	8.9	0.81	0.18	0.15
SvO2R	74.7	14.7	53.3	90.0	−0.62	73.6	11.3	7.8	8.5	0.91	0.15	0.14
ΔSavO2C	22.7	14.7	5.3	42.7	0.32	23.3	11.2	7.9	8.2	0.96	0.48	0.46
ΔSavO2R	8.0	14.7	−8.0	30.7	0.70	9.4	11.7	8.5	8.2	1.03	1.23	1.27
ΔSarO2C	17.0	11.0	4.0	32.0	0.32	17.4	8.4	5.9	6.2	0.96	0.48	0.46
ΔSarO2R	6.0	11.0	−6.0	23.0	0.70	7.1	8.8	6.4	6.2	1.03	1.23	1.27
fOERvC	0.3	0.2	0.1	0.5	0.24	0.24	0.10	0.07	0.07	1.00	0.47	0.47
fOERvR	0.1	0.2	−0.1	0.4	0.55	0.11	0.11	0.08	0.08	1.07	1.24	1.33
fOERrC	0.2	0.1	0.1	0.4	0.24	0.21	0.10	0.07	0.07	0.92	0.47	0.43
fOERrR	0.1	0.1	−0.1	0.3	0.55	0.08	0.10	0.07	0.08	1.01	1.24	1.25
PaCO2	47.0	10.4	36.4	62.9	0.78	48.1	8.2	6.2	6.0	1.03	0.17	0.18
PaO2	46.6	6.4	38.6	56.4	0.57	46.9	5.4	3.5	4.3	0.83	0.12	0.10
pH	7.4	0.1	7.2	7.5	−0.50	7.36	0.08	0.06	0.06	1.02	0.01	0.01
MABP	50.0	7.0	41.0	60.0	0.40	49.8	5.7	4.3	4.2	1.02	0.11	0.11
CVP	10.0	3.0	6.0	13.0	0.18	9.6	2.2	1.6	1.6	1.01	0.23	0.23
HR	173.0	18.0	150.0	195.0	−0.01	173	13.7	9.6	10.9	0.88	0.08	0.07
Hb	15.9	2.0	13.7	18.6	0.16	16.0	1.5	1.1	1.1	1.00	0.09	0.09

*Data were collected for 48 postoperative hours in 172 patients for a total of 7644 hourly measures*.

*Variables are summarized by P50,median; IQR, interquartile range; P5, 5^th^ percentile value; P95, P95^th^ percentile value; SK, skewness; SD, mean, standard deviation; SD-W, within-subject standard deviation; SD-B, between-subject standard deviation; SD, standard deviation; W/B, ratio of within-subject vs between-subject coefficient of variation; CV, coefficient of variation; CV(W), within-patient component of the coefficient of variation*.

### Temporal Trends

**Figure 1:**
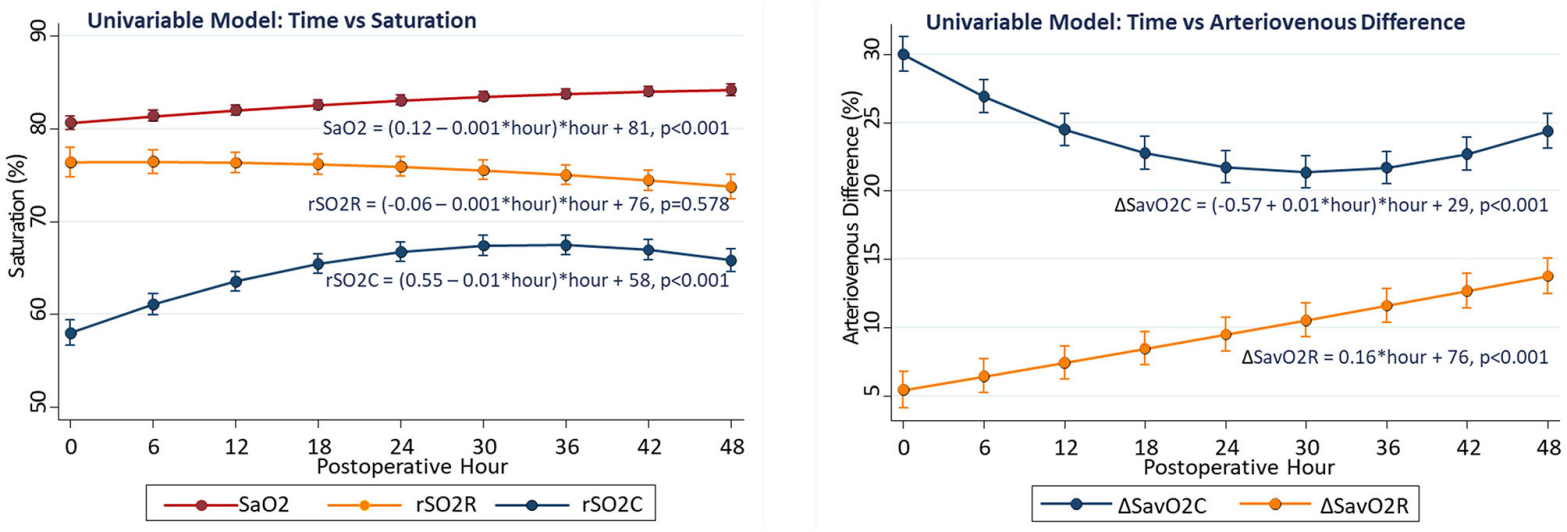
Changes in regional oxygenation and arteriovenous difference with time in univariable models.

Time-dependent changes in arterial and regional oxygenation were evident in univariable models. The SaO2 gradually increased over time (linear slope term 0.12 ± 0.01%/h, *p* < 0.001) in the early postoperative period. This increase was largely paralleled by the cerebral rSO2C (slope 0.55 ± 0.02%/h, *p* < 0.001), while the renal rSO2R did not change significantly (slope −0.01 ± 0.01%/h, *p* = 0.578). The time-dependent changes were opposite in direction for ΔSavO2C (−0.57 ± 0.03%/h, *p* < 0.001) vs. ΔSavO2R (+0.16 ± 0.03%/h, *p* < 0.001). These differential changes emphasize the importance of both the arterial oxygen content and regional vascular controls as determinants of regional oxygenation (see Figure 1).

### Univariable Models of PaCO2 Effects

**Figure 2:**
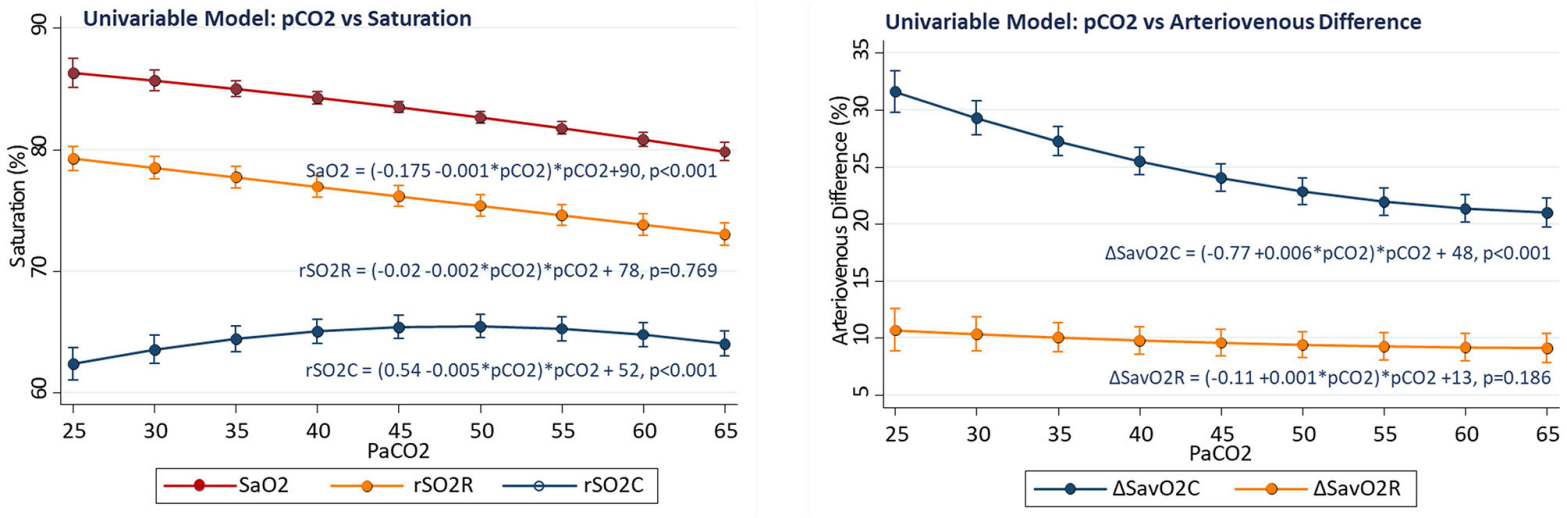
Changes in regional oxygenation and with arterial PaCO2 in univariable models.

Associations of regional measures with arterial PaCO2 were tested first in univariable models. There was positive association of cerebral rSO2C with PaCO2 (linear slope term = +0.54 ± 0.07%/torr, *p* < 0.001), while the relationship for somatic rSO2R with PaCO2 was not significant (slope−0.02 ± 0.07%/torr, *p* = 0.769), and the relationship for arterial saturation with PaCO2 was inverse (linear slope −0.175, *p* < 0.001). The relationship between PaCO2 and rSO2C also showed significant non-linearity, with maximal rSO2C occurring with PaCO2 in the 45–55 torr range. Over the PaCO2 range of 25–60 torr, the average rSO2C change was +12% (56.2 ± 1.2 to 68.8 ± 0.6, *p* < 0.001), while the average renal rSO2R change was −9% (80.8 ± 1.3 to 71.4 ± 0.8, *p* < 0.001).The slope change with PaCO2 was significantly negative for ΔSavO2C (slope −0.77 ± 0.10, *p* < 0.001), but not significant for ΔSavO2R (−0.11 ± 0.10, *p* = 0.186) The cerebral and renal regions showed distinctly different baseline patterns of saturation and blood flow, with the cerebral region showing lower saturation and wider arteriovenous difference over the range of PaCO2 compared to the renal region, despite differential changes with PaCO2 (see Figure 2).

### Time-Dependent PaCO2 Effects

**Figure 3:**
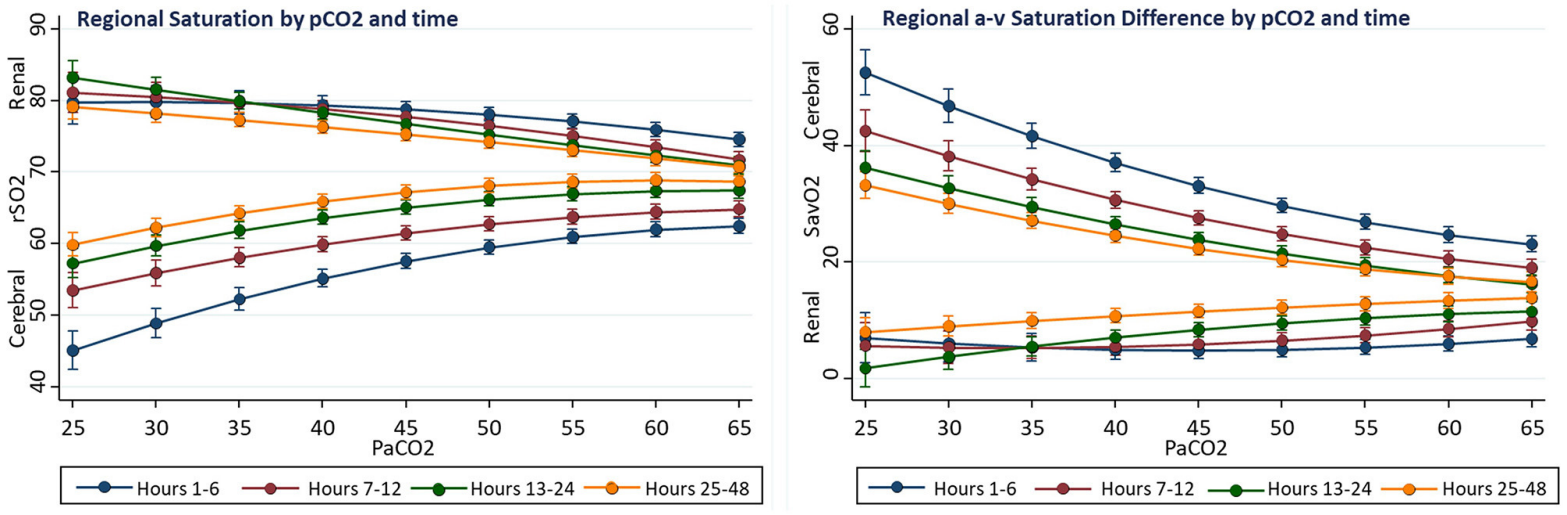
Interactions of time and PaCO2 on regional oxygenation and flow in univariable fixed-effect models. The effect on PaCO2 on cerebral rSO2C decreased with time, while the effect on renal rSO2R increased.

Complex interactions were observed between the effects of PaCO2 and time on regional oxygenation and flow measures. Compared to hours 24–48, the first 12 postoperative hours showed the average cerebral rSO2C was 7% lower (60.2 ± 0.2 vs. 67.6 ± 0.3, *p* < 0.001), renal rSO2R was 3% higher (77.6 ± 0.4 vs. 74.2 ± 0.4, *p* < 0.001), cerebral ΔSavO2C was 7% larger (29.1 ± 0.5 vs. 21.4 ± 0.4, *p* < 0.001), and renal ΔSavO2R was 7% smaller (5.9 ± 0.6 vs. 12.9 ± 0.5, *p* < 0.001). Compared to hours 24–48, the effect of PaCO2 over the range 25–60 torr during the first 12 postoperative hours on cerebral rSO2C was 6% greater (+15.1 ± 2.0 vs. +9.5 ± 1.5, *p* < 0.001), and the effect on renal rSO2 was 3% lower (−6.7 ± 2.4 vs. −10.4 ± 1.9, *p* < 0.036). During the first 12 postoperative hours, a PaCO2 > 55 torr was necessary to achieve a cerebral rSO2 of 60%, while this target could be met with PaCO2 of 40 torr after the first 24 h. Overall, the differences between PaCO2 effect on cerebral and renal regional measures was greater in the early postoperative period. A graphical summary is presented in Figure 3.

### Multivariable Models

Multivariable models were used to reveal the influence of individual factors on observed changes in regional oxygenation and blood flow as observed over time. The effects of PaCO2 and postoperative time were determined with a complex interaction expression to allow for non-linearity. Multiple factors had significant individual effects, some with differential effects for cerebral and renal regional measures. The known determinants of tissue oxygen delivery (SaO2, MABP, and Hb) all showed significant effects. For all primary dependent variables, there were significant independent effects of PaCO2 (alone and interacted with time) after controlling for these other factors. Neither shunt type nor survival status had influence on regional oxygenation or flow measures in multivariable models. The simplified model results are summarized in Table 4, and the complete models are shown in Supplementary Table S2.

**Table 4:** Multivariable regression results.

**Coefficient/ Model**	**rSO2C**	**rSO2R**	**ΔSavO2C**	**ΔSavO2R**
SaO2	0.190^***^ (0.0160)	0.203^***^ (0.0181)	1.080^***^ (0.0213)	1.063^***^ (0.0241)
MABP	0.160^***^ (0.0145)	0.165^***^ (0.0164)	−0.213^***^ (0.0194)	−0.220^***^ (0.0219)
Hb	0.673^***^ (0.0531)	0.904^***^ (0.0601)	−0.897^***^ (0.0708)	−1.205^***^ (0.0802)
PaCO2	0.989^***^ (0.143)	0.412^*^ (0.162)	−1.318^***^ (0.191)	−0.549^*^ (0.215)
Hour	0.457 (0.397)	1.775^***^ (0.443)	−0.610 (0.530)	−2.366^***^ (0.591)
((PaCO2) # (PaCO2))	−0.00650^***^ (0.00127)	−0.00464^**^ (0.00143)	0.00866^***^ (0.00169)	0.00619^**^ (0.00190)
(All & PaCO2)	0.982^***^ (0.142)	0.407^***^ (0.160)	−1.310^***^ (0.189)	−0.543^**^ (0.214)
(All & hour)	0.462 (0.390)	1.739^***^ (0.435)	−0.616 (0.520)	−2.318^***^ (0.580)
(All PaCO2 & hour)	1.454^***^ (0.498)	2.085^***^ (0.557)	−1.938^***^ (0.664)	−2.780^***^ (0.743)
Heart rate	−0.0396^***^ (0.00632)	−0.145^***^ (0.00713)	0.0528^***^ (0.00842)	0.193^***^ (0.00950)
CVP	−0.0896^*^ (0.0359)	−0.685^***^ (0.0407)	0.119^#x0002A;^ (0.0479)	0.913^***^ (0.0542)
Weight	0.0700 (0.838)	1.961^**^ (0.692)	−0.0934 (1.118)	−2.614^**^ (0.923)
Age	−0.627^***^ (0.0997)	−0.357^***^ (0.0825)	0.836^***^ (0.133)	0.476^***^ (0.110)
Gender (female)	−0.365 (0.905)	0.738 (0.748)	0.486 (1.207)	−0.984 (0.997)
CPB time	−0.0102 (0.00923)	−0.0203^**^ (0.00765)	0.0136 (0.0123)	0.0271^**^ (0.0102)
DHCA time	−0.143^*^ (0.0662)	−0.108^*^ (0.0548)	0.190^*^ (0.0883)	0.144^*^ (0.0731)
Shunt type (RVPA)	−0.00761 (0.898)	−0.700 (0.743)	0.0101 (1.198)	0.933 (0.991)
ACP+AAB	−1.679 (3.391)	7.507^**^ (2.798)	2.239 (4.522)	−10.01^**^ (3.731)
Survival	−2.052 (1.891)	−1.883 (1.586)	2.737 (2.521)	2.510 (2.114)
(constant)	7.543 (6.921)	55.72^***^ (6.717)	−10.06 (9.228)	−74.29^***^ (8.956)
N	7644	7606	7644	7606
R2 (overall)	0.211	0.356	0.203	0.371
R2 (between)	0.161	0.459	0.0564	0.382
R2 (within)	0.320	0.221	0.397	0.326
Rho	0.593	0.430	0.593	0.430

Coefficients are expressed as point estimates and (standard error), with significance designated at p <0.05 (*), p <0.01 (**), and p <0.001 (***).

*Non-linear effects were included for PaCO2, hour, and the interaction (#). Simplified coefficients combining non-linear and linear independent factors are presented for clarity (&) but the separate factors were used in regression models. The complete regression model parameters are shown in Supplemental Table S2*.

### Time-Dependent Multivariable Effects

**Figure 4:**
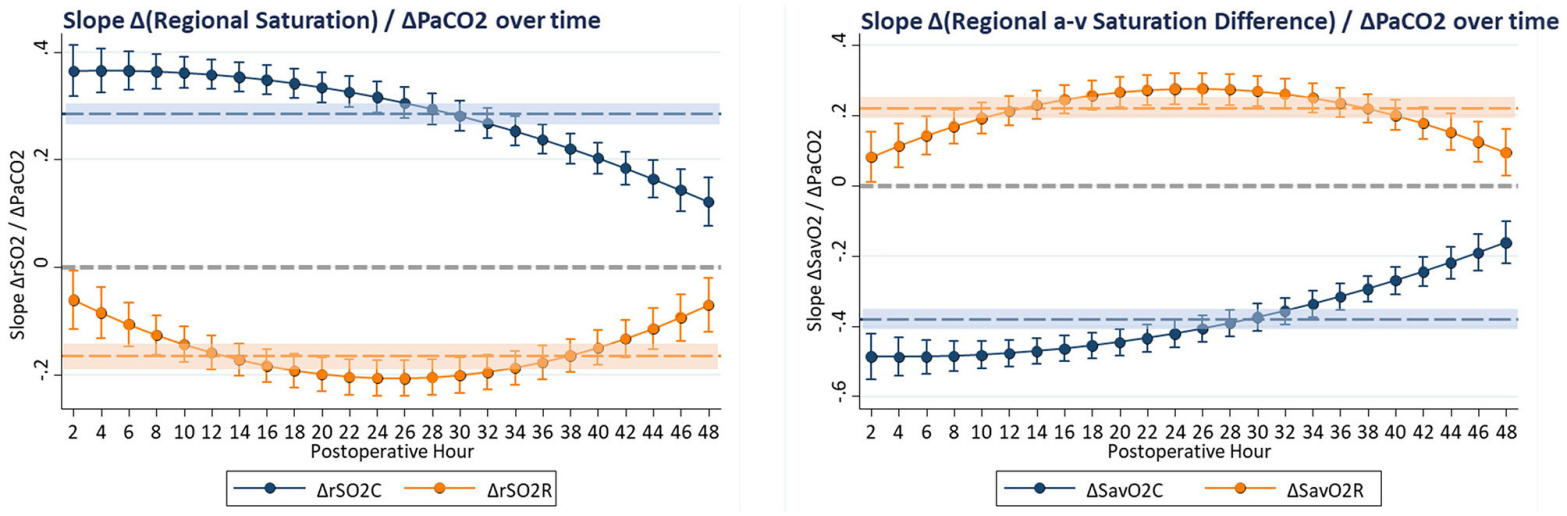
The slope of change in regional oxygenation or arteriovenous difference vs. change in PaCO2 was computed at hourly intervals from multivariable models. Covariates were constrained to clinically realistic values near the observed means (SaO2 = 83%, MABP = 51 mmHg, Hb = 15 gm/dl, CVP = 10 mmHg, weight = 3.2 kg, age = 8 days, CPB time = 172 min, and DHCA time = 20 min). These hourly coefficients are displayed graphically, showing effects of PaCO2 that change with time but have persistently differential effects on cerebral and renal circulations, with average effects shown as shaded regions.

**Figure 5:**
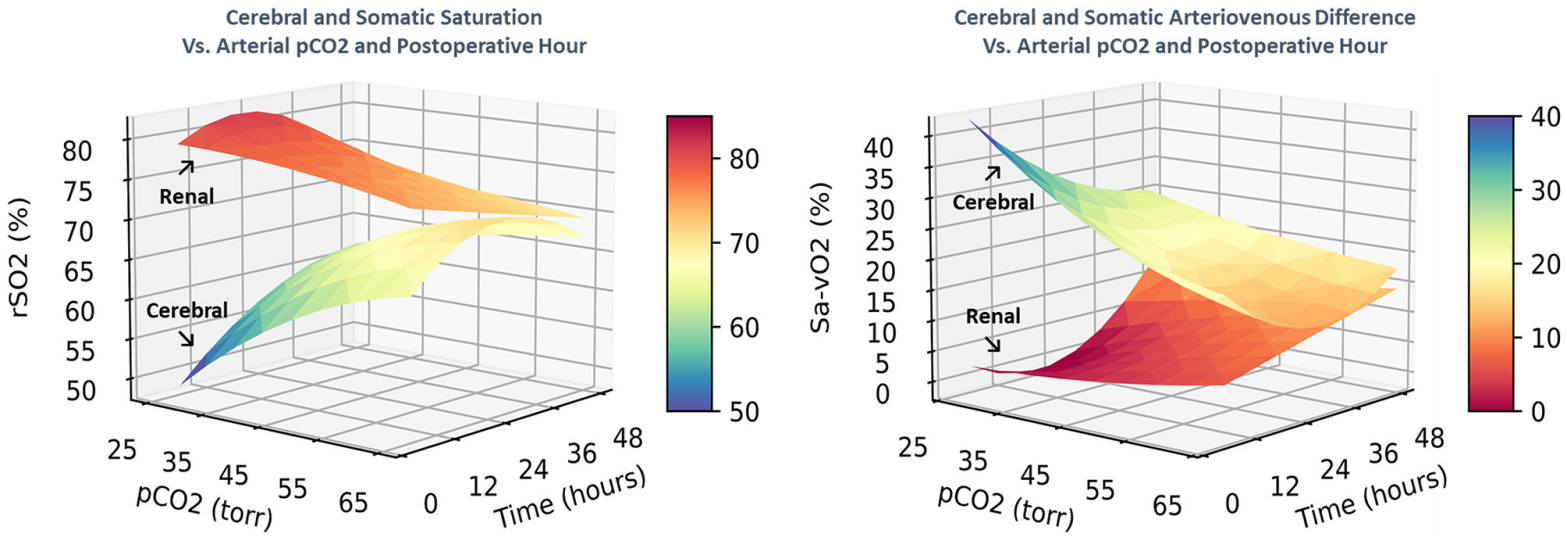
The combined effects of postoperative time and PaCO2 on regional saturation and arteriovenous difference are displayed as 3-dimensional surfaces. The differential circulatory controls on cerebral and renal circulatory beds are characterized by distinct spatial regions with different shapes.

These complex time-dependent non-linear effects of PaCO2 were simplified by computing the instantaneous slope of the change in regional saturation measure per unit change in PaCO2 at each hour with other covariates held constant at realistic clinical values near their observed means (SaO2 = 83%, MABP = 51 mmHg, Hb = 15 gm/dl, weight = 3.2 kg, and age = 8 days). The average expected response to a 1-unit increase in PaCO2 on cerebral rSO2C was positive (+0.350 ± 0.017, CI: 0.316 to 0.384, *p* < 0.001), while the expected response on renal rSO2R was negative (−0.220 ± 0.020, CI: −0.258 to −0.182, *p* < 0.001). For arteriovenous difference, the expected response on cerebral ΔSavO2C was negative (−0.467 ± 0.023, CI: −0.512 to −0.422, *p* < 0.001), while the response on renal ΔSavO2R was positive (+0.293 ± 0.026, CI: 0.242 to 0.344, *p* < 0.001). These expressions clarify the differential effects of changes in PaCO2 on regional circulations, with a change in PaCO2 consistently resulting in opposite effects on cerebral vs. renal circulations. The magnitude of these responses changed significantly over time, with cerebral effects highest in the first 18 postoperative hours, and renal effects reaching a maximum at 18–32 postoperative hours, as shown in Figure 4. The differences in both baseline oxygenation and time-dependent effects of PaCO2 are evident as distinct surface representations of regional circulations in Figure 5.

## Discussion

This study used NIRS to measure cerebral and renal/somatic oxygenation and blood flow following the Norwood procedure in nearly 200 neonates. Several novel observations were evident in this study. The major finding was the differential effect of changes in PaCO2 on cerebral and renal/somatic oxygenation and blood flow. These findings are consistent with known effect of increasing PaCO2 on cerebral vasodilation (34, 43, 44) and pulmonary vasoconstriction (45, 46), both of which might increase cerebral blood flow (47–50). However, the reduction in renal/somatic blood flow with increased PaCO2 suggests that the effects of PaCO2 on the pulmonary circulation were not predominant, and an alternative mechanism might be at work. If total systemic blood flow is relatively constant, then a reduction in cerebrovascular resistance with increased PaCO2 will divert blood flow from renal/somatic to the cerebral bed, consistent with the effects we observed. Our findings suggest that manipulation of PaCO2 is an intervention to alter the distribution of systemic blood flow by exploiting the differential sensitivity of cerebral and renal circulations to PaCO2, particularly when total systemic flow is limited.

The effects of CO2 exhibited significant time-dependence, with increased CO2 sensitivity of the cerebral circulation in the early postoperative period. This finding might be related to the pH-stat CPB management strategy that utilizes deliberate induction of extreme hypercapnia (PaCO2 80–100 torr) to counteract the leftward shift in oxyhemoglobin affinity during deep hypothermia. Regardless of duration of whole-body CPB, ACP, or DHCA, these patients were treated using pH-stat blood gas management strategies to maintain tissue oxygen tension during all phases of deep hypothermia. Hysteresis in the CO2-cerebrovascular resistance relationship (45) could therefore explain the observed shift over the first postoperative day. These observations suggest that targeted modulation of PaCO2 with deliberate hypercapnia in the early postoperative period is a physiologically rational strategy to normalize cerebral hemodynamics following significant exposure to pH-stat hypothermic CPB.

The observed relationships of Hb and SaO2 on regional oxygenation and blood flow were largely consistent with known effects and each contributed significantly to oxygenation in both cerebral and renal fields. The regression coefficient for SaO2 on both rSO2C and rSO2R was very close to the theoretical 25% arterial contribution to field saturation from the NIRS device calibration supporting the generalizability of the validation model (51–57). This empiric validation of the model also implies corresponding accuracy for derivation of regional venous saturation, arteriovenous difference, and fractional extraction by applying this model to synchronous SaO2 and rSO2 measures.

The differential relationship of MABP to cerebral and renal oxygenation deserves comments. Univentricular parallel circulation is characterized by an accentuated flow-resistance tradeoff in the systemic circulation, particularly in mesenteric and renal beds. Activation of the sympathetic nervous system with mesenteric/renal vasoconstriction is common following major operative intervention, contributing to systemic hypoperfusion, cellular hypoxia and shock even with preservation of blood pressure (40, 58, 59). We have previously described a potential flow-pressure tradeoff (60) and low cardiac output state utilizing renal NIRS (13) following the Norwood procedure with a more complex relationship on renal oxygenation described herein. Pressure autoregulation for cerebral circulation undergoes important developmental changes, and can be impaired by hypothermia and hypercapnia (37, 61–64). The positive association between MABP and rSO2C could be explained by MABP below a theoretical lower limit of autoregulation or with impairment of autoregulation by the above mechanisms. Cerebral pressure autoregulation is likely more continuously variable than absolute (26, 62, 65, 66).

Postnatal age showed a small but significant negative relationship with cerebral rSO2C, even though age at operation was relatively tightly constrained. This postnatal decrease in rSO2C has been previously described in healthy neonates, ascribed to an increase in cerebral metabolic rate following birth. These findings suggest potential advantage of operation at younger age. Although CPB time had no apparent effect, this may be related to potential protective effects of antegrade cerebral perfusion and alpha-adrenergic blockade. These effects were measurable in the renal circulation with associated increased rSO2R and lower ΔSavO2.

Although the RV-PA (Sano) shunt type has been associated with better early survival (67, 68) possibly related to improved myocardial oxygen supply-demand balance, the global hemodynamic effects of the RV-PA shunt are not clearly different from the MBT shunt, with very similar mean arterial pressure and saturations (69–71). Accordingly, we found no influence of shunt type on determinants of regional saturation or blood flow in multivariable models. Similarly, we did not a find a difference related to survival status, suggesting that regional circulator controls may not be important determinants of survival. However, this study cannot directly answer whether specific manipulation of PaCO2 to alter oxygenation and blood flow in regional circulations could also alter outcomes.

Like all patients with complete mixing of pulmonary and systemic venous blood, neonates with HLHS following a Norwood procedure will have significant hemoglobin desaturation in arterial blood, adding to risk of impaired systemic oxygen delivery, but limiting exposure of tissues to hyperoxic conditions, especially compared to normal term and preterm neonates (72–76). Although changes in both PaO2 and PaCO2 during re-oxygenation following birth and resuscitation have the potential to cause tissue injury through free radical pathways (77–80), this concern may have more relevance during cardiopulmonary bypass, ECMO support, or beyond the extremes of PaCO2 and PaO2 observed in this report (Table 3). Given the 5–95% intervals of PaCO2 (36–63 torr), PaO2 (38–56 torr), and SaO2 (75–91%), we would not expect significant effect of CO2 manipulation on tissue redox states or free radical generation. Both hypocapnia and hypercapnia have been associated with risk of cerebral injury in premature neonates, especially when rapid or extreme, with alteration in cerebral vascular resistance as a potential mechanism. This report describes the changes in both cerebral and renal-somatic blood flow and oxygenation associated with changes in PaCO2, and these regional NIRS measures would be thus rational targets for goal-directed ventilation strategies including dynamic manipulation of PaCO2.

The observational nature of this study is its major weakness. However, the population size and longitudinal data collection methods make the sample large enough for application of complex multivariable methods. Although multivariable multilevel and within-subject techniques were used to reduce bias and confounding, only limited causal association can be inferred. The population was restricted to patients with HLHS, undergoing Norwood repair utilizing deep hypothermic CPB, with known circulatory limitations. Although the findings are consistent with known physiologic mechanisms, caution should be observed when generalizing the observed effects to patients with other conditions and treatment strategies.

In summary, we found that arterial PaCO2 had differential effects on cerebral and renal circulation in neonates with HLHS following the Norwood procedure. The effects are measurable with a monitoring strategy that utilizes cerebral and somatic NIRS, thus facilitating individualized goal-directed interventions (81, 82).

## Data Availability Statement

The data analyzed in this study is subject to the following licenses/restrictions: a subset of the dataset will be available by contacting the corresponding author. Requests to access these datasets should be directed to George M. Hoffman, ghoffman@mcw.edu.

## Ethics Statement

The studies involving human participants were reviewed and approved by Children's Hospital of Wisconsin Institutional Review Board. Written informed consent to participate in this study was provided by the participants' legal guardian/next of kin.

## Author Contributions

GH was responsible for conception and design of the study, database maintenance, statistical analysis, and wrote the first draft of the manuscript. All authors contributed to manuscript revision, read, and approved the submitted version.

## Funding

This study was supported by institutional resources at Children's Hospital and Medical College of Wisconsin.

## Conflict of Interest

The authors declare that the research was conducted in the absence of any commercial or financial relationships that could be construed as a potential conflict of interest.

## Publisher's Note

All claims expressed in this article are solely those of the authors and do not necessarily represent those of their affiliated organizations, or those of the publisher, the editors and the reviewers. Any product that may be evaluated in this article, or claim that may be made by its manufacturer, is not guaranteed or endorsed by the publisher.

## References

[1] Binder C Urlesberger B Avian A Pocivalnik M Muller W Pichler G. Cerebral and peripheral regional oxygen saturation during postnatal transition in preterm neonates. J Pediatr. (2013) 163:394–9. 10.1016/j.jpeds.2013.01.026 23434123

[2] Pichler G Binder C Avian A Beckenbach E Schmolzer GM Urlesberger B. Reference ranges for regional cerebral tissue oxygen saturation and fractional oxygen extraction in neonates during immediate transition after birth. J Pediatr. (2013) 163:1558–63. 10.1016/j.jpeds.2013.07.007 23972642

[3] Hoffman GM Stuth EA Jaquiss RD Vanderwal PL Staudt SR Troshynski TJ. Changes in cerebral and somatic oxygenation during stage 1 palliation of hypoplastic left heart syndrome using continuous regional cerebral perfusion. J Thorac Cardiovasc Surg. (2004) 127:223–33. 10.1016/j.jtcvs.2003.08.021 14752434

[4] Hoffman GM Ghanayem NS Tweddell JS. Non-invasive monitoring of oxygen delivery. In: da Cruz EM, Ivy DD, Jaggers JJ, editors. Pediatric and Congenital Cardiology, Cardiac Surgery and Intensive Care. London: Springer-Verlag London Ltd (2014). p. 835–55. Available online at: http://link.springer.com/10.1007/978-1-4471-4619-3_105

[5] Tweddell JS Ghanayem NS Mussatto KA Mitchell ME Lamers LJ Musa NL. Mixed venous oxygen saturation monitoring after stage 1 palliation for hypoplastic left heart syndrome. Ann Thorac Surg. (2007) 84:1301–11. 10.1016/j.athoracsur.2007.05.047 17888987

[6] Barnea O Austin EH Richman B Santamore WP. Balancing the circulation: theoretic optimization of pulmonary/systemic flow ratio in hypoplastic left heart syndrome. J Am Coll Cardiol. (1994) 24:1376–81. 10.1016/0735-1097(94)90123-6 7523473

[7] Randsbaek F Riordan CJ Storey JH Montgomery WD Santamore WP Austin EH. Animal model of the univentricular heart and single ventricular physiology. J Invest Surg. (1996) 9:375–84. 10.3109/08941939609021279 8951661

[8] Ghanayem NS Hoffman GM Mussatto KA Frommelt MA Cava JR Mitchell ME. Perioperative monitoring in high-risk infants after stage 1 palliation of univentricular congenital heart disease. J Thorac Cardiovasc Surg. (2010) 140:857–63. 10.1016/j.jtcvs.2010.05.002 20621312

[9] Chakravarti SB Mittnacht AJ Katz JC Nguyen K Joashi U Srivastava S. Multisite near-infrared spectroscopy predicts elevated blood lactate level in children after cardiac surgery. J Cardiothorac Vasc Anesth. (2009) 23:663–7. 10.1053/j.jvca.2009.03.014 19447648

[10] Johnson BA Hoffman GM Tweddell JS Cava JR Basir M Mitchell ME. Near-infrared spectroscopy in neonates before palliation of hypoplastic left heart syndrome. Ann Thorac Surg. (2009) 87:571–7; discussion 577–9. 10.1016/j.athoracsur.2008.10.043 19161781

[11] Hoffman GM Brosig CL Mussatto KA Tweddell JS Ghanayem NS. Perioperative cerebral oxygen saturation in neonates with hypoplastic left heart syndrome and childhood neurodevelopmental outcome. J Thorac Cardiovasc Surg. (2013) 146:1153–64. 10.1016/j.jtcvs.2012.12.060 23317941

[12] Abu-Sultaneh S Hehir DA Murkowski K Ghanayem NS Liedel J Hoffmann RG. Changes in cerebral oxygen saturation correlate with s100b in infants undergoing cardiac surgery with cardiopulmonary bypass. Pediatr Crit Care Med. (2014) 15:219–28. 10.1097/PCC.0000000000000055 24366505

[13] Hoffman GM Ghanayem NS Scott JP Tweddell JS Mitchell ME Mussatto KA. Postoperative cerebral and somatic near-infrared spectroscopy saturations and outcome in hypoplastic left heart syndrome. Ann Thorac Surg. (2017) 103:1527–35. 10.1016/j.athoracsur.2016.09.100 28012642

[14] Hoffman GM Niebler RA Scott JP Bertrandt RA Wakeham MK Thompson NE. Interventions associated with treatment of low cardiac output after stage 1 norwood palliation. Ann Thorac Surg. (2021) 111:1620–7. 10.1016/j.athoracsur.2020.05.068 32652068

[15] Petrova A Mehta R. Near-infrared spectroscopy in the detection of regional tissue oxygenation during hypoxic events in preterm infants undergoing critical care. Pediatr Crit Care Med. (2006) 7:449–54. 10.1097/01.PCC.0000235248.70482.14 16885790

[16] Dittrich S Priesemann M Fischer T Boettcher W Muller C Alexi-Meskishvili V. Circulatory arrest and renal function in open-heart surgery on infants. Pediatr Cardiol. (2002) 23:15–9. 10.1007/s00246-001-0005-3 11922502

[17] Mittnacht AJ. Near infrared spectroscopy in children at high risk of low perfusion. Curr Opin Anaesthesiol. (2010) 23:342–7. 10.1097/ACO.0b013e3283393936 20421789

[18] Wernovsky G Kuijpers M Van Rossem MC Marino BS Ravishankar C Dominguez T. Postoperative course in the cardiac intensive care unit following the first stage of Norwood reconstruction. Cardiol Young. (2007) 17:652–65. 10.1017/S1047951107001461 17986364

[19] Uebing A Furck AK Hansen JH Nufer E Scheewe J Dütschke P. Perioperative cerebral and somatic oxygenation in neonates with hypoplastic left heart syndrome or transposition of the great arteries. J Thorac Cardiovasc Surg. (2011) 142:523–30. 10.1016/j.jtcvs.2011.01.036 21450312

[20] Dent CL Spaeth JP Jones B V. Schwartz SM Glauser TA Hallinan B. Brain magnetic resonance imaging abnormalities after the Norwood procedure using regional cerebral perfusion. J Thorac Cardiovasc Surg. (2006) 131:190–7. 10.1016/j.jtcvs.2005.10.003 16399311

[21] Kaufman J Almodovar MC Zuk J Friesen RH. Correlation of abdominal site near-infrared spectroscopy with gastric tonometry in infants following surgery for congenital heart disease. Pediatr Crit Care Med. (2008) 9:62–8. 10.1097/01.PCC.0000298640.47574.DA 18477915

[22] Skaryak LA Chai PJ Kern FH Greeley WJ Ungerleider RM. Blood gas management and degree of cooling: effects on cerebral metabolism before and after circulatory arrest. J Thorac Cardiovasc Surg. (1995) 110:1649–57. 10.1016/S0022-5223(95)70026-9 8523875

[23] Undar A Andropoulos DB Fraser J Kurth CD O'Rourke MM O'Hara IB. Comparison of pH-stat and alpha-stat cardiopulmonary bypass on cerebral oxygenation and blood flow in relation to hypothermic circulatory arrest in piglets (multiple letters). Anesthesiology. (1999) 90:926–7. 10.1097/00000542-199903000-00050 10078703

[24] Greeley WJ Kern FH Meliones JN Ungerleider RM. Effect of deep hypothermia and circulatory arrest on cerebral blood flow and metabolism. Ann Thorac Surg. (1993) 56:1464–6. 10.1016/0003-4975(93)90731-V 8267469

[25] Schell RM Kern FH Greeley WJ Schulman SR Frasco PE Croughwell ND. Cerebral blood flow and metabolism during cardiopulmonary bypass. Anesth Analg. (1993) 76:849–65. 10.1213/00000539-199304000-00029 8466029

[26] Smith B Vu E Kibler K Rusin C Easley RB Andropoulos D. Does hypothermia impair cerebrovascular autoregulation in neonates during cardiopulmonary bypass? Paediatr Anaesthesia. (2017) 27:905–10. 10.1111/pan.13194 28653463

[27] Taylor RH Burrows FA Bissonnette B. Cerebral pressure-flow velocity relationship during hypothermic cardiopulmonary bypass in neonates and infants. Anesth Analg. (1992) 74:636–42. 10.1213/00000539-199205000-00003 1567028

[28] Sood ED Benzaquen JS Davies RR Woodford E Pizarro C. Predictive value of perioperative near-infrared spectroscopy for neurodevelopmental outcomes after cardiac surgery in infancy. J Thorac Cardiovasc Surg. (2013) 145:435–8. 10.1016/j.jtcvs.2012.10.033 23219333

[29] Kussman BD Wypij D Laussen PC Soul JS Bellinger DC Dinardo JA. Relationship of intraoperative cerebral oxygen saturation to neurodevelopmental outcome and brain magnetic resonance imaging at 1 year of age in infants undergoing biventricular repair. Circulation. (2010) 122:245–54. 10.1161/CIRCULATIONAHA.109.902338 20606124 PMC2945235

[30] Sanchez-De-Toledo J Chrysostomou C Munoz R Lichtenstein S Sao-Avilés CA Wearden PD. Cerebral regional oxygen saturation and serum neuromarkers for the prediction of adverse neurologic outcome in pediatric cardiac surgery. Neurocrit Care. (2014) 21:133–9. 10.1007/s12028-013-9934-y 24203460

[31] Gist KM Kaufman J Da Cruz EM Friesen RH Crumback SL Linders M. A decline in intraoperative renal near-infrared spectroscopy is associated with adverse outcomes in children following cardiac surgery. Pediatr Crit Care Med. (2016) 17:342–9. 10.1097/PCC.0000000000000674 26914625 PMC5123446

[32] Owens GE King K Gurney JG Charpie JR. Low renal oximetry correlates with acute kidney injury after infant cardiac surgery. Pediatr Cardiol. (2011) 32:183–8. 10.1007/s00246-010-9839-x 21085945

[33] Scott JP Hoffman GM. Near-infrared spectroscopy: exposing the dark (venous) side of the circulation. Paediatr Anaesthesia. (2014) 24:74–88. 10.1111/pan.12301 24267637

[34] Ramamoorthy C Tabbutt S Kurth CD Steven JM Montenegro LM Durning S. Effects of inspired hypoxic and hypercapnic gas mixtures on cerebral oxygen saturation in neonates with univentricular heart defects. Anesthesiology. (2002) 96:283–8. 10.1097/00000542-200202000-00010 11818757

[35] Chock VY Ramamoorthy C Van Meurs KP. Cerebral autoregulation in neonates with a hemodynamically significant patent ductus arteriosus. J Pediatr. (2012) 160:936–42. 10.1016/j.jpeds.2011.11.054 22226574 PMC3335982

[36] Tabbutt S Ramamoorthy C Montenegro LM Durning SM Kurth CD Steven JM. Impact of inspired gas mixtures on preoperative infants with hypoplastic left heart syndrome during controlled ventilation. Circulation. (2001) 104(12 Suppl. 1):I159–I64. 10.1161/hc37t1.094818 11568049

[37] Chock VY Kwon SH Ambalavanan N Batton B Nelin LD Chalak LF. Cerebral oxygenation and autoregulation in preterm infants (early NIRS study). J Pediatr. (2020) 227:94–100.e1. 10.1016/j.jpeds.2020.08.036 32818482

[38] Tweddell JS Hoffman GM Mussatto KA Fedderly RT Berger S Jaquiss RDB. Improved survival of patients undergoing palliation of hypoplastic left heart syndrome : lessons learned from 115 consecutive patients. Circulation. (2002) 106(12 Suppl. 1):I82–I9. 10.1016/S1062-1458(02)01082-6 12354714

[39] Hoffman GM Scott JP Ghanayem NS Stuth EA Mitchell ME Woods RK. Identification of time-dependent risks of hemodynamic states after stage 1 norwood palliation. Ann Thorac Surg. (2020) 109:155–62. 10.1016/j.athoracsur.2019.06.063 31404548

[40] Anand KJ Hickey PR. Halothane-morphine compared with high-dose sufentanil for anesthesia and postoperative analgesia in neonatal cardiac surgery. N Engl J Med. (1992) 326:1–9. 10.1056/NEJM199201023260101 1530752

[41] Hoffman GM Ghanayem NS. Perioperative neuromonitoring in pediatric cardiac surgery: techniques and targets. Prog Pediatr Cardiol. (2010) 29:123–30. 10.1016/j.ppedcard.2010.06.006

[42] Kilpack VD Stayer SA McKenzie ED Fraser CD Jr Andropoulos DB. Limiting circulatory arrest using regional low flow perfusion. J Extra Corpor Technol. (2004) 36:133–8. 15334752

[43] Kety SS Schmidt CF. The effects of active and passive hyperventilation on cerebral blood flow, cerebral oxygen consumption, cardiac output, and blood pressure of normal young men. J Clin Invest. (1946) 25:107–19. 10.1172/JCI101680 21016304

[44] Kety SS Schmidt CF. The effects of altered arterial tensions of carbon dioxide and oxygen on cerebral blood flow and cerebral oxygen consumption of normal young men. J Clin Invest. (1948) 27:484–92. 10.1172/JCI101995 16695569 PMC439519

[45] Gordon JB Rehorst-Paea LA Hoffman GM Nelin LD. Pulmonary vascular responses during acute and sustained respiratory alkalosis or acidosis in intact newborn piglets. Pediatr Res. (1999) 46:735–41. 10.1203/00006450-199912000-00013 10590032

[46] Puybasset L Stewart T Rouby JJ Cluzel P Mourgeon E Belin MF. Inhaled nitric oxide reverses the increase in pulmonary vascular resistance induced by permissive hypercapnia in patients with acute respiratory distress syndrome. Anesthesiology. (1994) 80:1254–67. 10.1097/00000542-199406000-00013 8010472

[47] Li J Zhang G Holtby H Bissonnette B Wang G Redington AN. Carbon dioxide-a complex gas in a complex circulation: Its effects on systemic hemodynamics and oxygen transport, cerebral, and splanchnic circulation in neonates after the Norwood procedure. J Thorac Cardiovasc Surg. (2008) 136:1207–14. 10.1016/j.jtcvs.2008.02.096 19026805

[48] Fogel MA Durning S Wernovsky G Pollock AN Gaynor JW Nicolson S. Brain versus Lung: hierarchy of feedback loops in single-ventricle patients with superior cavopulmonary connection. Circulation. (2004) 110(11 Suppl.):II147–II52. 10.1161/01.CIR.0000138346.34596.99 15364854

[49] Hoskote A Li J Hickey C Erickson S Van Arsdell G Stephens D. The effects of carbon dioxide on oxygenation and systemic, cerebral, and pulmonary vascular hemodynamics after the bidirectional superior cavopulmonary anastomosis. J Am Coll Cardiol. (2004) 44:1501–9. 10.1016/j.jacc.2004.06.061 15464335

[50] Jobes DR Nicolson SC Steven JM Miller M Jacobs ML Norwood WI Jr. Carbon dioxide prevents pulmonary overcirculation in hypoplastic left heart syndrome. Ann Thorac Surg. (1992) 54:150–1. 10.1016/0003-4975(92)91166-7 1610228

[51] Booth E Dukatz C Ausman J Wider M. Cerebral and somatic venous oximetry in adults and infants. Surg Neurol Int. (2010) 1:75. 10.4103/2152-7806.73316 21170366 PMC2997227

[52] Abdul-Khaliq H Troitsch D Berger F Lange PE. Regional transcranial oximetry with near infrared spectroscopy (NIRS) in comparison with measuring oxygen saturation in the jugular bulb in infants and children for monitoring cerebral oxygenation. Biomed Eng. (2000) 45:328–32. 10.1515/bmte.2000.45.11.328 11155535

[53] Nagdyman N Fleck T Schubert S Ewert P Peters B Lange PE. Comparison between cerebral tissue oxygenation index measured by near-infrared spectroscopy and venous jugular bulb saturation in children. Intensive Care Med. (2005) 31:846–50. 10.1007/s00134-005-2618-0 15803294

[54] Pollard V Prough DS DeMelo AE Deyo DJ Uchida T Stoddart HF. Validation in volunteers of a near-infrared spectroscope for monitoring brain oxygenation *in vivo*. Anesth Analg. (1996) 82:269–77. 10.1097/00000539-199602000-00010 8561326

[55] Kim MB Ward DS Cartwright CR Kolano J Chlebowski S Henson LC. Estimation of jugular venous O_2_ saturation from cerebral oximetry or arterial O2 saturation during isocapnic hypoxia. J Clin Monit Comput. (2000) 16:191–9. 10.1023/a:1009940031063 12578103

[56] Watzman HM Kurth CD Montenegro LM Rome J Steven JM Nicolson SC. Arterial and venous contributions to near-infrared cerebral oximetry. Anesthesiology. (2000) 93:947–53. 10.1097/00000542-200010000-00012 11020744

[57] Sørensen H Secher NH Rasmussen P. A note on arterial to venous oxygen saturation as reference for NIRS-determined frontal lobe oxygen saturation in healthy humans. Front Physiol. (2014) 4:403. 10.3389/fphys.2013.00403 24478709 PMC3897873

[58] Bailey SM Hendricks-Muñoz KD Mally P. Splanchnic-cerebral oxygenation ratio as a marker of preterm infant blood transfusion needs. Transfusion. (2012) 52:252–60. 10.1111/j.1537-2995.2011.03263.x 21790634

[59] Anand KJ Sippell WG Schofield NM Aynsley-Green A. Does halothane anaesthesia decrease the metabolic and endocrine stress responses of newborn infants undergoing operation? Br Med J (Clin Res Ed). (1988) 296:668–72. 10.1136/bmj.296.6623.668 3128362 PMC2545294

[60] Hoffman GM Tweddell JS Ghanayem NS Mussatto KA Stuth EA Jaquis RDB. Alteration of the critical arteriovenous oxygen saturation relationship by sustained afterload reduction after the Norwood procedure. J Thorac Cardiovasc Surg. (2004) 127:738–45. 10.1016/S0022-5223(03)01315-1 15001902

[61] Bailey SM Hendricks-Munoz KD Mally P. Cerebral, renal, and splanchnic tissue oxygen saturation values in healthy term newborns. Am J Perinatol. (2014) 31:339–44. 10.1055/s-0033-1349894 23873114

[62] Rhee CJ da Costa CS Austin T Brady KM Czosnyka M Lee JK. Neonatal cerebrovascular autoregulation. Pediatr Res. (2018) 84:602–10. 10.1038/s41390-018-0141-6 30196311 PMC6422675

[63] Hoffman SB Cheng YJ Magder LS Shet N Viscardi RM. Cerebral autoregulation in premature infants during the first 96 hours of life and relationship to adverse outcomes. Arch Dis Childhood Fetal Neonatal Ed. (2019) 104:F473–F9. 10.1136/archdischild-2018-315725 30385514

[64] Alderliesten T Dix L Baerts W Caicedo A Van Huffel S Naulaers G. Reference values of regional cerebral oxygen saturation during the first 3 days of life in preterm neonates. Pediatr Res. (2016) 79:55–64. 10.1038/pr.2015.186 26389823

[65] Schopfer L Habre W Pichon I Fodor GH. Effect of permissive mild hypercapnia on cerebral vasoreactivity in infants: a randomized controlled crossover trial. Anesth Analg. (2021) 133:976–83. 10.1213/ANE.0000000000005325 33410612

[66] Brady KM Mytar JO Lee JK Cameron DE Vricella LA Thompson WR. Monitoring cerebral blood flow pressure autoregulation in pediatric patients during cardiac surgery. Stroke. (2010) 41:1957–62. 10.1161/STROKEAHA.109.575167 20651273 PMC5498798

[67] Ohye RG Sleeper LA Mahony L Newburger JW Pearson GD Lu M. Comparison of shunt types in the Norwood procedure for single-ventricle lesions. N Engl J Med. (2010) 362:1980–92. 10.1056/NEJMoa0912461 20505177 PMC2891109

[68] Si MS Pearson GD Ohye RG. Shunt choice in single right ventricle patients: an update. Expert Rev Cardiovasc Therapy. (2013) 11:1691–700. 10.1586/14779072.2013.847790 24215198

[69] Frommelt PC Sheridan DC Mussatto KA Hoffman GM Ghanayem NS Frommelt MA. Effect of shunt type on echocardiographic indices after initial palliations for hypoplastic left heart syndrome: Blalock-Taussig shunt versus right ventricle-pulmonary artery conduit. J Am Soc Echocardiogr. (2007) 20:1364–73. 10.1016/j.echo.2007.04.009 17604955

[70] Ghanayem NS Jaquiss RDB Cava JR Frommelt PC Mussatto KA Hoffman GM. Right ventricle-to-pulmonary artery conduit versus Blalock-Taussig shunt: a hemodynamic comparison. Ann Thorac Surg. (2006) 82:1603–9; discussion 1609–10. 10.1016/j.athoracsur.2006.05.103 17062213

[71] Mroczek T Małota Z Wójcik E Nawrat Z Skalski J. Norwood with right ventricle-to-pulmonary artery conduit is more effective than Norwood with Blalock-Taussig shunt for hypoplastic left heart syndrome: mathematic modeling of hemodynamics. Eur J Cardio Thorac Surg. (2011) 40:1412–8. 10.1016/j.ejcts.2011.03.033 21546259

[72] Dawson JA Omar Kamlin CF Vento M Wong C Cole TJ Donath SM. Defining the reference range for oxygen saturation for infants after birth. Pediatrics. (2010) 125:1340–7. 10.1542/peds.2009-1510 20439604

[73] Askie LM Darlow BA Davis PG Finer N Stenson B Vento M. Effects of targeting lower versus higher arterial oxygen saturations on death or disability in preterm infants. Cochrane Database Syst Rev. (2017) 4:CD011190. 10.1002/14651858.CD011190.pub2 28398697 PMC6478245

[74] Askie LM Darlow BA Finer N Schmidt B Stenson B Tarnow-Mordi W. Association between oxygen saturation targeting and death or disability in extremely preterm infants in the neonatal oxygenation prospective meta-analysis collaboration. JAMA. (2018) 319:2190–201. 10.1001/jama.2018.5725 29872859 PMC6583054

[75] Oei JL Kapadia V Rabi Y Saugstad OD Rook D Vermeulen MJ. Neurodevelopmental outcomes of preterm infants after randomisation to initial resuscitation with lower (FiO 2 < 0.3) or higher (FiO 2 > 0.6) initial oxygen levels. An individual patient meta-analysis. Arch Dis Childhood Fetal Neonatal Ed. (2021) 10.1136/archdischild-2021-321565 34725105

[76] Mathias M Chang J Perez M Saugstad O. Supplemental oxygen in the newborn: historical perspective and current trends. Antioxidants. (2021) 10:1879. 10.3390/antiox10121879 34942982 PMC8698336

[77] Jakkula P Reinikainen M Hästbacka J Pettilä V Loisa P Karlsson S. Targeting low- or high-normal Carbon dioxide, Oxygen, and Mean arterial pressure After Cardiac Arrest and REsuscitation: study protocol for a randomized pilot trial. Trials. (2017) 18:507. 10.1186/s13063-017-2257-0 29084585 PMC5663085

[78] Humaloja J Vento M Kuligowski J Andersson S Piñeiro-Ramos JD Sánchez-Illana Á. High oxygen does not increase reperfusion injury assessed with lipid peroxidation biomarkers after cardiac arrest: a *post hoc* analysis of the COMACARE trial. J Clin Med. (2021) 10:4226. 10.3390/jcm10184226 34575337 PMC8471647

[79] Millán I Piñero-Ramos JD Lara I Parra-Llorca A Torres-Cuevas I Vento M. Oxidative stress in the newborn period: useful biomarkers in the clinical setting. Antioxidants. (2018) 7:193. 10.3390/antiox7120193 30558164 PMC6316621

[80] Law BHY Asztalos E Finer NN Yaskina M Vento M Tarnow-Mordi W. Higher versus lower oxygen concentration during respiratory support in the delivery room in extremely preterm infants: a pilot feasibility study. Children. (2021) 8:942. 10.3390/children8110942 34828655 PMC8625238

[81] Harer MW Chock VY. Renal tissue oxygenation monitoring-an opportunity to improve kidney outcomes in the vulnerable neonatal population. Front Pediatr. (2020) 8:241. 10.3389/fped.2020.00241 32528917 PMC7247835

[82] Chock VY Variane GFT Netto A Van Meurs KP. NIRS improves hemodynamic monitoring and detection of risk for cerebral injury: cases in the neonatal intensive care nursery. J Matern Fetal Neonatal Med. (2020) 33:1802–10. 10.1080/14767058.2018.1528223 30244630

