# Supplementary material for: Effects of Arterial Carbon Dioxide Tension on Cerebral and Somatic Regional Tissue Oxygenation and Blood Flow in Neonates After the Norwood Procedure With Deep Hypothermic Cardiopulmonary Bypass
Source: Front Pediatr. 2022 Feb 11;10:762739. doi: 10.3389/fped.2022.762739 (PMC8873518; doi:10.3389/fped.2022.762739)
Supplement: Supplementary file 1 [file Data_Sheet_1.pdf]

Supplemental Data

Table S1: Patient population demographics, operative characteristics, and outcomes

Characteristic	*All (N=195)*	*Excluded (N=17)*	*Analysis Set (N=178)*	*P-value (for difference)*
Weight at S1P (kg)	3.20 (0.70) (2.90; 3.60)	3.00 (0.75) (2.60; 3.35)	3.20 (0.70) (2.90; 3.60)	0.052
Age at S1P (days)	7.00 (4.0) (5.00; 9.00)	8.00 (5.0) (6.00; 11.00)	7.00 (4.0) (5.00; 9.00)	0.196
Gender male	111 (57)	7 (41)	104 (58)	0.170
female	92 (43)	10 (59)	74 (42)	
Gestational Age (weeks)	38 (1) (38,39)	36 (4.5) (35,39.5)	38 (1) (38,39)	0.290
CPB Time (minutes)	171.0 (51.0) (151.0; 202.0)	258.0 (150.0) (212.5; 362.5)	168.0 (43.5) (148.8; 192.0)	<0.001
DHCA Time (minutes)	13.0 (10.0) (8.0; 18.0)	18.0 (16.5) (11.0; 27.5)	12.0 (8.0) (8.0; 16.0)	0.009
Shunt Type MBTS	103 (53%)	9 (53%)	94 (53%)	0.992
RVPA	92 (47%)	8 (47%)	84 (47%)	
ECMO Support Yes	20 (10%)	14 (82%)	6 (3%)	<0.001
No	175 (90%)	3(18%)	172 (97%)	
Operative Survival	176 (90%)	9 (53%)	167 (94%)	<0.001
Died	19 (10%)	8 (47%)	11 (6%)	

Data are median (interquartile range and interval or count (percent). Data were excluded from analysis during ECMO and operative support, which continued from the operating room through the entire 48 hour analysis period for 17 patients, yielding an analysis set of 178 patients. Differences between patients included and excluded from analysis were summarized by significance testing. Patients excluded from analysis due to continuous ECMO support were slightly smaller and had longer operative support times and lower survival.

Table S2: Multivariable regression results

Coefficient / Model	*rSO2Cc*	*rSO2R*	*ΔSavO2C*	*ΔSavO2R*
SaO2	0.190 ***	0.203 ***	1.080 ***	1.063 ***
	(0.0160)	(0.0181)	(0.0213)	(0.0241)
MABP	0.160 ***	0.165 ***	-0.213 ***	-0.220 ***
	(0.0145)	(0.0164)	(0.0194)	(0.0219)
Hb	0.673 ***	0.904 ***	-0.897 ***	-1.205 ***
	(0.0531)	(0.0601)	(0.0708)	(0.0802)
PaCO2	0.989 ***	0.412 *	-1.318 ***	-0.549 *
	(0.143)	(0.162)	(0.191)	(0.215)
Hour	0.457	1.775 ***	-0.610	-2.366 ***
	(0.397)	(0.443)	(0.530)	(0.591)
((PaCO2) # (PaCO2))	-0.00650 ***	-0.00464 **	0.00866 ***	0.00619 **
	(0.00127)	(0.00143)	(0.00169)	(0.00190)
(All & PaCO2)	0.982 ***	0.407 ***	-1.310 ***	-0.543 **
	(0.142)	(0.160)	(0.189)	(0.214)
((Hour) # (Hour))	0.00414	-0.0361 ***	-0.00552	0.0481 ***
	(0.00805)	(0.00878)	(0.0107)	(0.0117)
(All & Hour)	0.462	1.739 ***	-0.616	-2.318 ***
	(0.390)	(0.435)	(0.520)	(0.580)
((PaCO2) # (Hour))	0.0104	-0.0628 ***	-0.0138	0.0837 ***
	(0.0153)	(0.0170)	(0.0204)	(0.0227)
((PaCO2^2) # (Hour))	-0.0000961	0.000511 **	0.000128	-0.000682 **
	(0.000146)	(0.000163)	(0.000194)	(0.000217)
((PaCO2) # (Hour^2))	-0.000523	0.00116 ***	0.000698	-0.00154 ***
	(0.000316)	(0.000346)	(0.000422)	(0.000461)
((PaCO2^2) # (Hour^2))	0.00000411	-0.00000926 **	-0.00000547	0.0000123 **
	(0.00000308)	(0.00000338)	(0.00000411)	(0.00000450)
(All PaCO2 & Hour)	1.454 ***	2.085 ***	-1.938 ***	-2.780 ***
	(0.498)	(0.557)	(0.664)	(0.743)
Heart Rate	-0.0396 ***	-0.145 ***	0.0528 ***	0.193 ***
	(0.00632)	(0.00713)	(0.00842)	(0.00950)
CVP	-0.0896 *	-0.685 ***	0.119 *	0.913 ***
	(0.0359)	(0.0407)	(0.0479)	(0.0542)
Weight	0.0700	1.961 **	-0.0934	-2.614 **
	(0.838)	(0.692)	(1.118)	(0.923)
Age	-0.627 ***	-0.357 ***	0.836 ***	0.476 ***
	(0.0997)	(0.0825)	(0.133)	(0.110)
Gender (female)	-0.365	0.738	0.486	-0.984
	(0.905)	(0.748)	(1.207)	(0.997)
CPB Time	-0.0102	-0.0203 **	0.0136	0.0271 **
	(0.00923)	(0.00765)	(0.0123)	(0.0102)
DHCA time	-0.143 *	-0.108 *	0.190 *	0.144 *
	(0.0662)	(0.0548)	(0.0883)	(0.0731)
Shunt type (RVPA)	-0.00761	-0.700	0.0101	0.933
	(0.898)	(0.743)	(1.198)	(0.991)

ACP+AAB	-1.679	7.507 **	2.239	-10.01 **
	(3.391)	(2.798)	(4.522)	(3.731)
Survival	-2.052	-1.883	2.737	2.510
	(1.891)	(1.586)	(2.521)	(2.114)
(constant)	7.543	55.72 ***	-10.06	-74.29 ***
	(6.921)	(6.717)	(9.228)	(8.956)
N	7644	7606	7644	7606
R2 (overall)	0.211	0.356	0.203	0.371
R2 (between)	0.161	0.459	0.0564	0.382
R2 (within)	0.320	0.221	0.397	0.326
Rho	0.593	0.430	0.593	0.430

Coefficients are expressed as point estimates and (standard error), with significance designated at $p<0.05$ (*), $p<0.01$ (**), and $p<0.001$ (***)

Non-linear effects were included for $PaCO_2$, hour, and the interaction (#). Simplified coefficients combining non-linear and linear independent factors are presented for clarity (&) but the separate factors were used in regression models.
